# A lifestyle-derived risk score for chronic atrophic gastritis: development and validation in 21,008 Chinese adults

**DOI:** 10.3389/fpubh.2026.1784898

**Published:** 2026-04-21

**Authors:** Runze Guo, Rui Zhang, Rong Jia, Jianjun Wu

**Affiliations:** School of Public Health, Gansu University of Chinese Medicine, Lanzhou, China

**Keywords:** chronic atrophic gastritis, gastric cancer prevention, lifestyle, prediction model, risk score, screening population

## Abstract

**Background:**

Chronic atrophic gastritis (AG) is a key precancerous lesion of gastric cancer. Modifiable lifestyle factors such as smoking, alcohol consumption, and specific dietary patterns are associated with the risk of AG. However, lifestyle indicators have not been integrated into clinical practice, and there is a lack of practical integrated risk assessment tools for primary healthcare settings.

**Objective:**

Develop and validate a simple lifestyle risk score to identify individuals at high risk of AG in the Chinese screening population.

**Methods:**

This population-based case–control study utilized data from the Upper Gastrointestinal Cancer Screening Program in Gansu Province, China. A total of 21,008 participants aged 40–70 years were included. AG status was determined based on endoscopic and pathological diagnoses. A risk score (range 0–7) was developed incorporating seven modifiable lifestyle factors, with fried food and garlic intake included as exploratory components based on prior literature. Associations were assessed using multivariate logistic regression, and predictive performance was evaluated through ROC curve analysis, calibration, and decision curve analysis.

**Results:**

The prevalence of AG in the total sample was 44.5%. A strong positive association was observed between the lifestyle risk score and AG risk (*P* for trend <0.001). After adjusting for age and sex, each one-point increase in the score was associated with a 51% increase in AG risk (adjusted OR = 1.506, 95% CI: 1.460–1.554). Compared with the low-risk group (score 0–1), the high-risk group (score ≥3) showed a significantly elevated AG risk (aOR = 3.614, 95% CI: 3.289–3.971). The area under the ROC curve for the score was 0.597 (95% CI: 0.590–0.604). The model demonstrated good calibration, and decision curve analysis indicated that applying this score in clinical decision-making provided net clinical benefit across a wide range of threshold probabilities (approximately 10–60%).

**Conclusion:**

This study developed and validated a simple lifestyle risk score that demonstrated a significant association with AG risk and modest but clinically useful predictive performance suitable for primary healthcare settings. This tool is applicable for rapid identification of high-risk AG individuals in primary healthcare settings and gastric cancer screening programs, providing practical evidence to guide targeted preventive interventions.

## 1 Introduction

Gastric cancer poses a significant global public health burden, particularly in East Asia (1, 2). Chronic atrophic gastritis (AG) is recognized internationally as a key precancerous lesion in gastric carcinogenesis. The dynamic progression from chronic inflammation to irreversible mucosal atrophy, intestinal metaplasia, dysplasia, and ultimately invasive carcinoma—known as the “Correa cascade”—illustrates the pathogenic sequence of gastric cancer development (3–5). Therefore, early identification and intervention at the reversible precancerous stage of AG represent a fundamental strategy for halting malignant progression and reducing the incidence and mortality of gastric cancer (4, 6).

Substantial evidence indicates that various modifiable lifestyle factors play important roles in the development and progression of AG. Smoking directly damages gastric mucosal DNA through carcinogens such as polycyclic aromatic hydrocarbons and nitrosamines present in tobacco (7). Alcohol consumption and its metabolite acetaldehyde disrupt the gastric mucosal barrier and increase its permeability (8). Dietary factors are also critical: high intake of salted and nitrite-rich pickled foods, low consumption of potentially protective garlic, and frequent consumption of fried foods rich in advanced glycation end products and oxidative stress agents have been associated with increased AG risk in studies; meanwhile, fresh vegetables and fruits—abundant in antioxidant vitamins, phytochemicals, and dietary fiber—have demonstrated clear protective effects (9–11).

However, most existing studies have examined individual risk factors in isolation, lacking an integrated assessment of cumulative exposure risks at the individual level (12). In resource-limited primary healthcare and population screening settings, there is a need for a simple, rapid, low-cost, and easy-to-use risk assessment tool that integrates multiple modifiable factors to enable accurate identification of high-risk individuals (13). This approach has gained consensus in prevention and screening for various chronic conditions, including cardiovascular disease (14), diabetes (15), and colorectal cancer (16).

Therefore, this study aimed to develop and validate a lifestyle-based risk score for AG using data from a large Chinese screening population. We hypothesized that this score would exhibit a dose–response relationship with AG risk and demonstrate good discriminative ability, calibration, and clinical utility. The findings are expected to provide an easily disseminable screening and health management tool for primary prevention of gastric cancer.

## 2 Data and methods

### 2.1 Study design and population

This study was conducted within the framework of the National Upper Gastrointestinal Cancer Early Detection Program in Wuwei City, one of the high-risk areas for upper gastrointestinal cancer in Gansu Province. Participants were recruited from high-risk individuals in 21 administrative villages across Jinyang, Siba, and Qingyuan Towns in Liangzhou District, Wuwei City. From November 2015 to November 2016, a health factor survey for upper gastrointestinal tumors was conducted in Gansu Province (following the 2014 edition of the Chinese Technical Guidelines for Cancer Screening, Early Diagnosis, and Treatment). Upper gastrointestinal endoscopy and pathological examinations were performed at the Gansu Wuwei Tumor Hospital as part of gastric cancer screening and health examinations. The study protocol was approved by the Ethics Committee of the National Cancer Center, Chinese Academy of Medical Sciences (Approval No.: 2015SQ00223), and all participants provided written informed consent prior to enrollment.

### 2.2 Study participants

Inclusion criteria: (1) aged 40–70 years (determined based on the Chinese Technical Guidelines for Cancer Screening, Early Diagnosis, and Treatment (2014 edition), which designates this range as the core high-risk population for upper gastrointestinal cancer screening in China; epidemiologically, AG incidence rises markedly after 40 years old, and individuals over 70 years old are excluded due to reduced endoscopy/follow-up feasibility); (2) completed a standardized questionnaire survey; (3) underwent upper gastrointestinal endoscopy with pathological biopsy; (4) had a definitive diagnosis of AG.

Exclusion criteria: (1) missing data on key lifestyle variables; (2) previously diagnosed with malignant lesions such as gastric cancer or gastric intraepithelial neoplasia; (3) history of gastric cancer surgery; (4) recent use of medications affecting *Helicobacter pylori* detection.

A total of 21,008 participants were ultimately included in the analysis, comprising 9,354 AG cases and 11,654 controls.

### 2.3 Variable definitions and measurements

#### 2.3.1 Outcome variable

According to the new Sydney standard, 5 tissue specimens were collected from each patient for pathological examination. The location and requirements for tissue collection were as follows: A1: small curvature of the gastric antrum (2–3 cm from pylorus); A2: large curvature of the gastric antrum (2–3 cm from pylorus); B1: small curvature of gastric body (4 cm from gastric angle); B2: large curvature of the gastric body (8 cm from cardia); IA: gastric angle. Biopsy tissue was fixed in formalin and sent to pathology. Routine paraffin sections were prepared and stained with hematoxylin–eosin (HE). The diagnosis of AG in this study was based on pathological diagnosis.

#### 2.3.2 Predictive variables (components of the risk score)

Based on literature review and expert consensus, seven modifiable lifestyle factors were selected to construct the risk score. Each factor was assigned 1 point if the risk condition was present:

Smoking: Current smoker (yes = 1 point).

Alcohol consumption: Current drinker (yes = 1 point).

High-frequency intake of pickled foods: Participants reporting “daily” or “frequent” consumption of pickled foods (e.g., salted vegetables, preserved meat) in the questionnaire (yes = 1 point).

Low-frequency garlic intake: Participants reporting “occasional” or “never” consumption of garlic (yes = 1 point).

High-frequency fried food intake: Participants reporting “daily” or “frequent” consumption of fried foods (yes = 1 point).

Low-frequency fresh vegetable intake: Participants reporting “occasional” or “never” consumption of fresh vegetables (yes = 1 point).

Low-frequency fresh fruit intake: Participants reporting “occasional” or “never” consumption of fresh fruits (yes = 1 point).

Total score calculation: The scores of the seven items were summed, ranging from 0 to 7. Based on the total score, participants were categorized into three risk levels: low risk (0–1 points), intermediate risk (2 points), and high risk (≥3 points).

Note: Fried food intake and low garlic intake were included as exploratory components based on extensive literature evidence supporting their potential roles in gastric carcinogenesis, despite not showing independent statistical significance in this specific population. Their retention allows for cross-study comparability and hypothesis generation for future research.

#### 2.3.3 Covariates

The following potential confounding factors were adjusted for in the analysis: age (continuous variable), sex, number of household members, quartiles of annual household income, body mass index (BMI), marital status, ethnicity, and source of drinking water.

### 2.4 Statistical analysis

#### 2.4.1 Descriptive analysis

Categorical variables were described using frequencies (percentages), with between-group comparisons performed using the Chi-square test. Continuous variables were expressed as mean ± standard deviation, and between-group comparisons were conducted using the Student’s *t*-test.

#### 2.4.2 Score association analysis

Multivariable logistic regression models were employed. Initially, crude odds ratios (ORs) were calculated. Subsequently, two adjusted models were constructed: Model 1 was adjusted for age and sex; Model 2 was further adjusted for covariates including number of household members, income, and BMI. A test for trend was performed by treating the lifestyle score as a continuous variable. Adjusted ORs (aORs) for different risk categories, compared to the low-risk group, were calculated.

#### 2.4.3 Predictive performance evaluation

Discrimination: Receiver operating characteristic (ROC) curves were plotted, and the area under the curve (AUC) along with its 95% confidence interval (CI) was calculated. The optimal risk cutoff was identified by maximizing the Youden index.

Calibration: The agreement between predicted probabilities and observed outcomes was assessed using the Brier score.

Clinical Utility: Decision curve analysis (DCA) was performed to evaluate the net benefit of applying the score for clinical decision-making across various threshold probabilities.

#### 2.4.4 Subgroup and sensitivity analyses

To assess the robustness and heterogeneity of the associations, comprehensive subgroup analyses were conducted. Subgroup variables included demographic characteristics (sex, age, and income), clinical features (BMI, family history of gastric cancer), and lifestyle factors (smoking, alcohol consumption, and garlic intake). All subgroup analyses utilized multivariable logistic regression models with appropriate adjustments for confounding factors. Differences between subgroups were evaluated by comparing the 95% CIs of the aORs.

Sensitivity analyses were also performed to evaluate the robustness of the findings through the following methods: (1) using different score thresholds to define the high-risk group; (2) developing a simplified score by excluding statistically non-significant items; (3) employing the *E*-value to assess the potential impact of unmeasured confounding on the primary results.

All statistical analyses were conducted using R software (version 4.2.5). A two-sided *p*-value < 0.05 was considered statistically significant.

## 3 Results

### 3.1 Characteristics of the study population

A total of 21,008 participants aged 40–70 years were ultimately included in this study, comprising 9,354 chronic atrophic gastritis (AG) cases (44.5%) and 11,654 controls (55.5%). The baseline characteristics of the study population are presented in Table 1.

**Table 1:** Baseline characteristics of the study population.

Variable	Control group *N* = 11,654^1^	Case group *N* = 9,354^1^	*p*-value^2^
Age (years)	52.19(7.77)	52.25(7.44)	0.581
Age group			<0.001
<50 years	5,647(48%)	4,424(47%)	
50–59 years	3,804(33%)	3,288(35%)	
60–70 years	2,203(19%)	1,642(18%)	
Sex			0.043
Male	5,698(49%)	4,441(47%)	
Female	5,956(51%)	4,913(53%)	
Household size	4.58(1.55)	4.40(1.56)	<0.001
Annual household income (¥)	48,963.24(39,520.15)	39,815.05(28,221.62)	<0.001
Income quartile			<0.001
Q1 (Low)	3,328(29%)	4,290(46%)	
Q2	2,169(19%)	1,861(20%)	
Q3	4,017(34%)	2,394(26%)	
Q4 (High)	2,140(18%)	809(8.6%)	
BMI (kg/m^2^)	23.69(2.98)	24.05(3.13)	<0.001
BMI category			<0.001
Underweight	345(3.0%)	235(2.5%)	
Normal	6,256(54%)	4,559(49%)	
Overweight	4,192(36%)	3,571(38%)	
Obese	861(7.4%)	989(11%)	
Lifestyle score			<0.001
0	2,660(23%)	1,705(18%)	
1	4,894(42%)	2,964(32%)	
2	3,074(26%)	2,950(32%)	
3	817(7.0%)	1,381(15%)	
4	199(1.7%)	328(3.5%)	
5	10(<0.1%)	26(0.3%)	
Risk category			<0.001
Low	7,554(65%)	4,669(50%)	
Medium	3,074(26%)	2,950(32%)	
High	1,026(8.8%)	1,735(19%)	
Smoking (High-risk)			<0.001
No	7,868(68%)	6,033(64%)	
Yes	3,786(32%)	3,321(36%)	
Alcohol consumption (High-risk)			<0.001
No	10,774(92%)	8,006(86%)	
Yes	880(7.6%)	1,348(14%)	
High-frequency pickled food			<0.001
No	11,351(97%)	8,758(94%)	
Yes	303(2.6%)	596(6.4%)	
Low-frequency garlic intake			0.500
No	6,109(52%)	4,948(53%)	
Yes	5,545(48%)	4,406(47%)	
High-frequency fried food			0.341
No	10,929(94%)	8,741(93%)	
Yes	725(6.2%)	613(6.6%)	
Low-frequency vegetable intake			0.004
No	11,570(99%)	9,251(99%)	
Yes	84(0.7%)	103(1.1%)	
Low-frequency fruit intake			<0.001
No	8,638(74%)	5,292(57%)	
Yes	3,016(26%)	4,062(43%)	
Primary water source			<0.001
Cellar water	3,238(28%)	5,064(54%)	
Lake water	1(<0.1%)	2(<0.1%)	
Deep well water	5,319(46%)	2,389(26%)	
Tap water	3,096(27%)	1,899(20%)	
Family history of any cancer	1,638(14%)	2,148(23%)	<0.001
Family history of GI cancer	1,228(11%)	1,664(18%)	<0.001

^1^Mean (SD); *n*(%).

^2^Welch Two Sample *t*-test; Pearson’s Chi-squared test; NA.

The average age of the case group was 52.25 ± 7.44 years, and that of the control group was 52.19 ± 7.77 years (*p* = 0.581), but there was a significant difference in age distribution (*p* < 0.001). The proportion of the 50–59 age group in the case group (35.2%) was slightly higher than that in the control group (32.6%). In terms of gender distribution, the proportion of females in the case group (52.5%) was slightly higher than that in the control group (51.1%, *p* = 0.043).

Regarding socioeconomic factors, the average family size of the case group (4.40 ± 1.56) was significantly smaller than that of the control group (4.58 ± 1.55, *p* < 0.001), and the family economic status was significantly worse. The average annual household income of the case group (39,815.05 ± 28,221.62 yuan) was significantly lower than that of the control group (48,963.24 ± 39,520.15 yuan, *p* < 0.001). Income stratification showed that the proportion of low-income (Q1) individuals in the case group was as high as 45.9%, significantly higher than 28.6% in the control group; while the proportion of high-income (Q4) individuals was only 8.6%, significantly lower than 18.4% in the control group (*p* < 0.001).

In terms of clinical indicators, the average BMI of the case group (24.05 ± 3.13 kg/m^2^) was significantly higher than that of the control group (23.69 ± 2.98 kg/m^2^, *p* < 0.001). The combined proportion of overweight and obesity in the case group was 48.8%, higher than 43.4% in the control group (*p* < 0.001). Blood pressure measurement showed that the systolic blood pressure of the case group was lower, but the diastolic blood pressure was higher.

Among the seven components of the lifestyle risk score, the case group had significantly higher positive rates in smoking (35.5% vs. 32.5%, *p* < 0.001), drinking (14.4% vs. 7.6%, *p* < 0.001), frequent intake of pickled foods (6.4% vs. 2.6%, *p* < 0.001), low-frequency vegetable intake (1.1% vs. 0.7%, *p* = 0.004), and low-frequency fruit intake (43.4% vs. 25.9%, *p* < 0.001) compared to the control group. There was no significant difference in the intake of fried foods and garlic between the two groups.

Overall, the average lifestyle risk score of the case group (1.97 ± 0.87) was significantly higher than that of the control group (1.67 ± 0.83, *p* < 0.001). By risk level, the proportion of high-risk individuals (≥3 points) in the case group was 18.6%, significantly higher than 8.8% in the control group; while the proportion of low-risk individuals (0–1 point) was 49.9%, significantly lower than 64.8% in the control group (*p* < 0.001).

In other factors, the proportion of individuals drinking well water (underground water storage facilities) in the case group (54.1%) was significantly higher than that in the control group (27.8%), while the proportion of those drinking deep well water and tap water was lower (*p* < 0.001). The proportion of individuals with a family history of tumors and digestive tract cancers in the case group was also significantly higher than that in the control group.

### 3.2 Distribution of lifestyle risk score and its association with AG prevalence

The distribution of the lifestyle risk score in the study population and its association with the prevalence of chronic atrophic gastritis (AG) are presented in Table 2. The scores ranged from 0 to 6 points, with the most prevalent score being 1 point (37.4%, 7,858/21,008) followed by 2 points (28.7%, 6,024/21,008). Based on the predefined risk categories, 58.1% of participants were classified as low-risk (0–1 points, 12,223/21,008), 28.7% as intermediate-risk (2 points), and 13.2% as high-risk (≥3 points).

**Table 2:** Distribution of lifestyle risk score and its association with AG prevalence.

Score	Risk category	Control group *n*(%)	AG case group *n*(%)	Total *n*	AG prevalence (%)
0	Low risk	2,660(60.9)	1705(39.1)	4,365	39.1
1	Low risk	4,894(62.3)	2,964(37.7)	7,858	37.7
2	Medium risk	3,074(51.0)	2,950(49.0)	6,024	49
3	High risk	817(37.2)	1,381(62.8)	2,198	62.8
4	High risk	199(37.8)	328(62.2)	527	62.2
5	High risk	10(27.8)	26(72.2)	36	72.2
6	High risk	1(100.0)	0(0.0)	1	0.0
Total		11,654	9,354	21,008	44.5

Categorical variables are presented as *n*(%). Risk category definitions: Low risk (0–1 points), medium risk (2 points), high risk (≥3 points).

The prevalence of AG demonstrated a significant and clear dose–response relationship with increasing lifestyle risk scores (*P* for trend <0.001). In the low-risk population, the AG prevalence slightly decreased from 39.1% at 0 points to 37.7% at 1 point. However, starting from the intermediate-risk category (2 points), the prevalence increased sharply to 49.0%. Among high-risk individuals, the prevalence further escalated: 62.8% at 3 points, 62.2% at 4 points, and 72.2% at 5 points. Only one participant had a score of 6 points, who belonged to the control group with an AG prevalence of 0%.

When analyzed by risk category, the AG prevalence was 38.3% (4,669/12,223) in the low-risk group (0–1 points), 49.0% (2,950/6,024) in the intermediate-risk group (2 points), and 62.8% (1,735/2,761) in the high-risk group (≥3 points). Compared with the low-risk group, the high-risk group showed a 64.0% relative increase in AG prevalence.

This clear gradient indicates that cumulative exposure to adverse lifestyle factors is significantly associated with AG risk, exhibiting a dose–response relationship. A score of 2 points (intermediate-risk) represents a significant turning point where the prevalence exceeds 50%, while a score of ≥3 points (high-risk) identifies a very high-risk population with an AG prevalence exceeding 60%.

### 3.3 Multivariate analysis of the association between lifestyle risk score and AG risk

Multivariable logistic regression analysis confirmed a significant association between the lifestyle risk score and the risk of chronic atrophic gastritis (Table 3). When the score was treated as a continuous variable, the unadjusted model showed that each 1-point increase in the lifestyle score was associated with a 36% increase in AG risk (OR = 1.363, 95% CI: 1.326–1.401, *p* < 0.001). After adjusting for age and sex, this association was strengthened, with each 1-point increase corresponding to a 51% increase in AG risk (aOR = 1.506, 95% CI: 1.460–1.554, *p* < 0.001). Further adjustment for additional covariates, including the number of household members, annual household income, BMI, and source of drinking water, slightly attenuated the association strength, but it remained highly significant (aOR = 1.487, 95% CI: 1.440–1.535, *p* < 0.001), indicating the robustness of the association between the lifestyle score and AG risk.

**Table 3:** Multivariable analysis of the association between lifestyle risk score and AG risk.

Model	OR	95%CI	*P*-value
Continuous score (per 1-point increase)			
Unadjusted model	1.363	1.326–1.401	<0.001
Adjusted for age and sex	1.506	1.460–1.554	<0.001
Adjusted for additional covariates*	1.487	1.440–1.535	<0.001
Risk category (Reference: Low risk)			
Medium risk vs. Low risk	1.794	1.679–1.916	<0.001
High risk vs. Low risk	3.614	3.289–3.971	<0.001

*Additional covariates included household size, annual household income, BMI, and primary water source.

Analysis by risk category revealed that compared with the low-risk group (0–1 points), the intermediate-risk group (2 points) had a significantly increased risk of developing AG, with a 79% higher risk (aOR = 1.794, 95% CI: 1.679–1.916, *p* < 0.001). The high-risk group (≥3 points) exhibited an even more pronounced increase in AG risk, being 3.6 times higher than that of the low-risk group (aOR = 3.614, 95% CI: 3.289–3.971, *p* < 0.001). A clear dose–response relationship was observed between risk categories and AG risk.

Sensitivity analyses using different cutoffs to define the high-risk group yielded consistent results (Supplementary Table 1). When defining high-risk using a cutoff of ≥1 point, the high-risk group had a 1.40-fold increased AG risk compared to the low-risk group (0 points) (95% CI: 1.310–1.488, *p* < 0.001). Using a cutoff of ≥2 points, the AG risk of the high-risk group increased to 2.14 times that of the low-risk group (95% CI: 2.021–2.274, *p* < 0.001). When defining high-risk with a cutoff of ≥3 points, the AG risk was the highest, being 2.73 times that of the low-risk group (95% CI: 2.527–2.944, *p* < 0.001). These results further confirm the robustness of the lifestyle score and its strong association with AG risk.

### 3.4 Analysis of the independent contributions of individual components of the lifestyle score

To further evaluate the independent associations between each component of the lifestyle risk score and the risk of chronic atrophic gastritis, we analyzed the seven lifestyle factors individually after adjusting for age and sex (Table 4).

**Table 4:** Association between individual lifestyle score components and AG risk (adjusted for age and sex).

Score component	aOR	95%CI	*P*-value
Smoking	1.478	1.357–1.611	<0.001
Alcohol consumption	2.342	2.129–2.576	<0.001
High-frequency pickled food consumption	2.553	2.217–2.939	<0.001
High-frequency fried food consumption	1.056	0.945–1.181	0.333
Low-frequency vegetable intake	1.528	1.144–2.041	0.004
Low-frequency fruit intake	2.208	2.083–2.341	<0.001
Low-frequency garlic intake	0.979	0.927–1.034	0.451

(1) All models were adjusted for age and sex. aOR = adjusted odds ratio; CI = confidence interval. (2) Although fried food and garlic intake did not show statistically significant independent associations in this population, they were retained as exploratory components in the main score based on prior evidence and their potential role as effect modifiers (see Discussion).

### 3.5 Evaluation of the predictive performance of the lifestyle risk score

**Figure 1:**
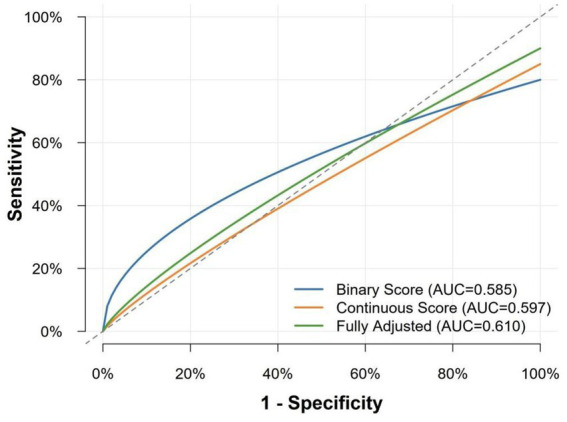
Receiver operating characteristic (ROC) curves comparing the predictive performance of the binary score, continuous score, and fully adjusted model.

To assess the discriminatory predictive ability of the lifestyle risk score for chronic atrophic gastritis (AG), this study plotted Receiver Operating Characteristic (ROC) curves and calculated the Area Under the Curve (AUC). As shown in Figure 1, the AUC values for the Binary Score, Continuous Score, and Fully Adjusted Model were 0.585, 0.597, and 0.610, respectively. The results indicate that the discriminatory performance of the lifestyle score for AG risk was low to moderate, with the Fully Adjusted Model showing slightly better discrimination than the other two versions, but overall not reaching the conventional threshold for a good predictive model (AUC ≥ 0.7).

For the Binary Score model, the optimal risk cutoff was determined to be 2 points by maximizing Youden’s index (Table 5). At this cutoff, the sensitivity of the lifestyle score was 50.1%, and the specificity was 64.8%. This means the score correctly identified 50.1% of AG cases and correctly classified 64.8% of non-cases as low-risk. At this point, the positive predictive value (PPV) was 53.3%, indicating that among individuals classified as high-risk (score ≥2 points), 53.3% actually had AG; the negative predictive value (NPV) was 61.8%, indicating that among those classified as low-risk (score <2 points), 61.8% were truly AG-free. The overall accuracy of the score was 58.3%, with a Youden’s index of 0.149.

**Table 5:** Predictive performance metrics (based on ROC curve analysis).

Metric	Value
Discrimination metrics	
AUC (95%CI)	0.597(0.590–0.604)
Performance at optimal cut-off (2 points)	
Sensitivity	50.1%
Specificity	64.8%
Positive predictive value (PPV)	53.3%
Negative predictive value (NPV)	61.8%
Accuracy	58.3%
Youden’s Index	0.149
Brier score	0.239

In terms of calibration assessment, the Brier score was 0.239, indicating acceptable agreement between the predicted probabilities and the observed AG prevalence (Table 5).

**Figure 2:**
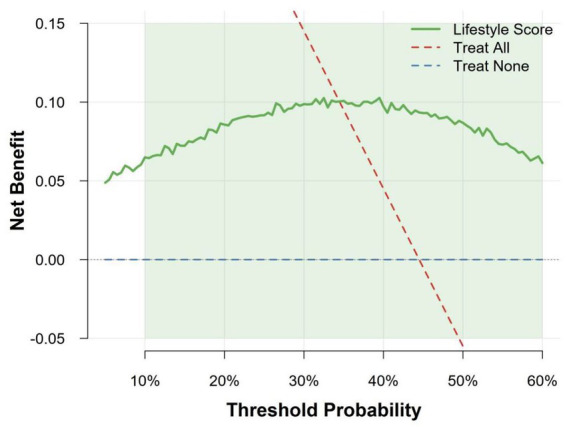
Decision curve analysis comparing the net clinical benefit of intervention based on the lifestyle score versus “treat all” and “treat none” strategies.

Decision curve analysis (Figure 2) demonstrated that across a wide range of threshold probabilities (approximately 10 to 60%), applying the lifestyle risk score to guide clinical decisions (e.g., deciding whether to perform gastroscopy) provided net clinical benefit compared to strategies of “intervention for all participants” or “no intervention for all participants.” This suggests practical utility of the score in real-world clinical scenarios.

### 3.6 Subgroup and sensitivity analyses

**Figure 3:**
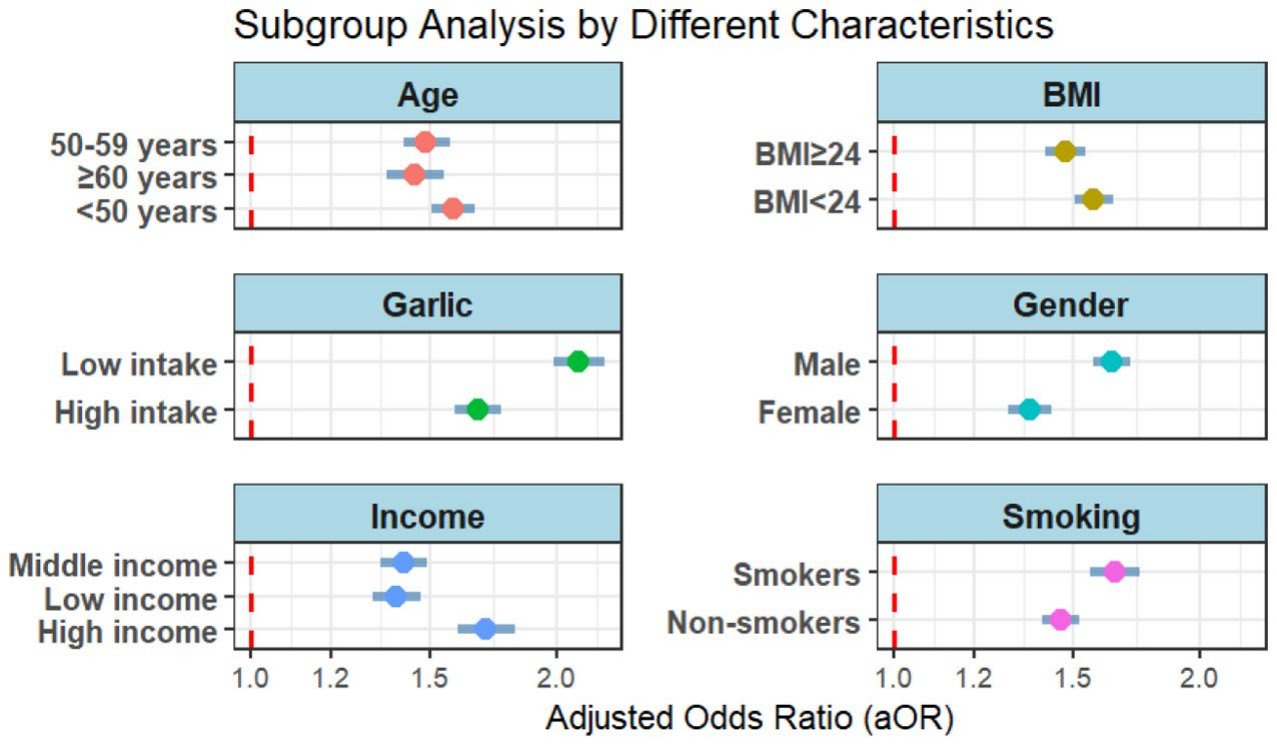
Subgroup analysis based on age, BMI, garlic intake, gender, income, and smoking status. Detailed subgroup analyses, including finer stratifications of income and BMI, are available upon request.

Sensitivity analyses demonstrated that the positive association between the lifestyle risk score and AG risk remained consistent across all subgroups (all *p* < 0.001), indicating the robustness of the association. To systematically evaluate the heterogeneity of the association, comprehensive subgroup analyses were performed (Figure 3). Although the strength of the association varied somewhat across subgroups, the 95% confidence intervals for all subgroups were greater than 1.0.

Figure 3 (forest plot of subgroup analysis) visually presents the adjusted odds ratios (aORs) stratified by sex, age, income, BMI, smoking, and garlic intake. Notably, the subgroup analysis validated the validity of individual components within the lifestyle score: the aOR was higher in smokers (aOR = 1.647) than in non-smokers (aOR = 1.458), and significantly higher in individuals with low-frequency garlic intake (aOR = 2.100) than in those with high-frequency intake (aOR = 1.674), providing empirical support for the weighting of the smoking and garlic items in the scoring system. The association strength was higher in males (aOR = 1.628) than in females (aOR = 1.355), and was strongest in individuals aged ≤50 years (aOR = 1.576).

To test the robustness of the primary results, a series of sensitivity analyses were conducted (detailed results are shown in Supplementary Tables 1–3). Using different cut-offs (≥1, ≥2, and ≥3 points) to define the high-risk group, the associations with AG remained highly significant (Supplementary Table 1). A simplified 5-item score, constructed by excluding non-significant items (fried food and low-frequency garlic intake), showed a stronger association with AG (aOR = 1.968), and its AUC slightly increased to 0.622 (Supplementary Table 2). A 3-item score comprising only the three core risk factors (smoking, alcohol consumption, and pickled food intake) demonstrated the strongest association (aOR = 2.297, AUC = 0.633). *E*-value analysis indicated that to explain the observed effect (aOR = 1.506), an unmeasured confounder with a relative risk of 2.38 would be required, suggesting that the observed association is unlikely to be entirely explained by unmeasured confounding (Supplementary Table 3).

## 4 Discussion

Using data from 21,008 Chinese adults, we developed and validated a seven-item lifestyle-based risk score for chronic atrophic gastritis (AG). Key findings include: (1) a robust dose–response relationship, with high-risk (score ≥3) individuals showing 3.6-fold higher AG risk than low-risk; (2) modest but acceptable discrimination (AUC = 0.585–0.610) for a lifestyle-only tool, with good calibration (Brier = 0.239) and clinically meaningful net benefit across 10–60% threshold probabilities; (3) strongest independent associations for pickled food (aOR = 2.55), alcohol (aOR = 2.34), and low fruit intake (aOR = 2.21), while fried food and garlic intake were not independently significant.

Our study integrated seven lifestyle factors related to gastric mucosal health, selected based on literature and expert consensus. The results align with numerous previous studies but also present noteworthy differences. This study confirmed smoking, alcohol consumption, and high-frequency pickled food intake as independent risk factors for AG, consistent with existing evidence (17). Pickled food (aOR = 2.55) and alcohol (aOR = 2.34) showed strong associations, highlighting the central role of high dietary salt/nitrite exposure and ethanol metabolites in chronic gastric mucosal injury (18). The strong protective association indicated by the absence of low-frequency fruit intake (aOR = 2.21) reinforces the importance of dietary antioxidants and phytochemicals in gastric mucosal protection (19). However, the lack of significant independent associations for high-frequency fried food and low-frequency garlic intake differs from some previous studies (20). These null findings likely reflect measurement limitations and population-specific factors: for fried food, crude frequency assessment and low exposure (6.4%) (21–23); for garlic, minimal consumption variability (97.4% low-frequency) and unmeasured *H. pylori* interaction (24–27). Notably, subgroup analysis revealed that the lifestyle score-AG association was significantly stronger in low-frequency garlic consumers (aOR = 2.100 vs. 1.674), suggesting garlic may act as an effect modifier (28). These findings highlight that lifestyle effects may be population-specific and measurement-dependent (29), considerations essential for score optimization and cross-population validation.

As exploratory components of the score, although high-frequency fried food intake and low-frequency garlic intake did not show statistically significant independent associations with AG in this study, they were retained in the main score for three critical reasons. First, garlic intake may act as an effect modifier rather than an independent risk factor: our subgroup analysis revealed that the association between the overall lifestyle score and AG risk was 25.4% stronger in low-frequency garlic consumers (aOR = 2.100) compared to high-frequency consumers (aOR = 1.674). This finding suggests that insufficient garlic intake amplifies the harmful effects of other adverse lifestyle factors on gastric mucosa, supporting its inclusion as a component that captures synergistic risk. Second, the non-significant association for fried food intake is likely attributable to methodological limitations rather than absence of biological effect. Our simple frequency-based assessment could not capture critical exposure dimensions such as frying oil type (e.g., repeated use of oil), frying temperature, or concentrations of specific carcinogenic compounds formed during frying (e.g., acrylamide, polycyclic aromatic hydrocarbons) (21–23). Additionally, the low exposure rate (6.4%) in this population may have resulted in insufficient statistical power. Third, both factors are well-documented in previous epidemiological studies to be associated with gastric mucosal health (20, 24, 25); their inclusion ensures the comprehensiveness of the score as a tool for assessing modifiable lifestyle risk factors and facilitates cross-study comparisons. We explicitly note these two factors as exploratory components of the score, and their optimal weighting may require further refinement in future multi-center studies with more detailed dietary assessments.

Although the discriminatory ability (AUC = 0.597) fell below the conventional threshold (AUC ≥ 0.7), this is expected for lifestyle-based models given the complex etiology of AG involving genetic, environmental, infectious, and lifestyle factors (30–32). By deliberately including only intervenable lifestyle factors, the score prioritizes preventive applicability over predictive precision. Lifestyle effects are inherently modest and cumulative—reflected in individual item aORs (~2.5) versus strong risk factors like *H. pylori*—consistent with other chronic disease models (e.g., type 2 diabetes, cardiovascular disease) where AUCs typically range 0.6–0.7 (33–36). Thus, AUC alone inadequately captures the value of this modifiable risk-focused tool.

The core value of this study lies not in pursuing ultimate predictive precision, but in providing a highly feasible risk stratification strategy for real-world primary care settings. Decision curve analysis (DCA) provides key evidence for this: within the 10–60% probability range typically considered by clinical decision-makers, using this score to guide the decision for gastroscopy yielded a net benefit superior to the extreme strategies of “screen all” or “screen none.” In a screening population with an AG prevalence of 44.5%, this implies that using the score can effectively optimize resource allocation by prioritizing limited gastroscopy resources for high-risk groups, thereby maximizing health benefits at the population level. Furthermore, all items in the score are derived from a short questionnaire, and the calculation involves a simple summation of scores (0–7), allowing assessment to be completed within 1 min by primary care physicians, community health workers, or even individuals. This simplicity is a key advantage for its potential large-scale adoption and integration into routine health check-ups and community screenings.

Large-scale subgroup analyses (*N* = 21,008) confirmed the score’s robustness across all predefined strata (all *p* < 0.001), with stronger associations in males, smokers, and low-frequency garlic consumers—supporting its applicability in targeted interventions.

Despite the demonstrated practical potential of this score, its limitations must be acknowledged. First, the most prominent and critical limitation is the lack of external validation. All participants were recruited from a single regional population in Gansu Province, China, with unique local dietary patterns (e.g., high consumption of pickled foods, drinking cellar water) that may affect the score’s generalizability to other ethnic/regional populations. Second, as a case–control study, the design itself cannot establish causality. Third, lifestyle data relied on self-reporting, which is susceptible to recall bias and social desirability bias. Fourth, despite adjusting for multiple covariates (household size, income, BMI, water source, etc.) and conducting E-value analysis (Supplementary Table 3), residual confounding cannot be entirely excluded, particularly regarding unmeasured dietary details and socioeconomic nuances. Fifth, the study did not adjust for *Helicobacter pylori* (*H. pylori*) infection, a well-recognized core risk factor for AG. *H. pylori* infection status was not fully measured in this study due to the absence of standardized detection data (e.g., 13C urea breath test, histopathology) for all participants. The lack of *H. pylori* adjustment substantially limits the causal interpretation of the association between lifestyle factors and AG, as *H. pylori* infection may interact with smoking, pickled food intake and other lifestyle factors to exacerbate gastric mucosal damage. Sixth, the study did not evaluate micronutrient deficiencies (e.g., vitamin B12, iron, folate) or restrictive dietary patterns (vegetarian/vegan status), which are known to play important roles in gastric mucosal health (37–39). The absence of these variables was due to the lack of detailed dietary nutrient assessment and dietary pattern classification in the original screening questionnaire, leading to the exclusion of these important variables that may introduce residual confounding.

Based on the above findings and limitations, future research could proceed in the following directions:

(1) Rigorous external validation and generalizability assessment: Validate the calibration and discrimination of the score in independent prospective cohorts, particularly multi-center cohorts with diverse dietary cultures, and explore the potential need to adjust items or weights based on population characteristics.

(2) Exploring model optimization pathways: Sensitivity analyses in this study have provided clues. The simplified (5-item, AUC = 0.622) and core (3-item, AUC = 0.633) versions showed optimization potential. Future work could explore optimizing item selection and weight assignment using machine learning algorithms. Hybrid models could also be developed, retaining the simplicity of this score while selectively integrating one or two low-cost, easily obtainable objective indicators (e.g., age, sex, or even serum pepsinogen ratio from finger-prick blood tests), aiming to improve predictive performance without substantially increasing complexity.

(3) Assessing long-term predictive value and intervention effects: Investigate whether the score can predict the progression of AG to more advanced lesions (intestinal metaplasia, dysplasia). More importantly, design and implement intervention studies based on this score to evaluate whether targeted lifestyle guidance or enhanced screening for high-risk individuals can effectively reduce AG incidence or delay its progression.

(4) Promoting tool translation and implementation science: Develop the score into user-friendly digital tools (e.g., mobile apps, WeChat mini-programs) or simple screening cards, and study the implementation barriers, cost-effectiveness, and acceptance among healthcare providers and patients in real-world primary care settings.

## 5 Conclusion

This study developed and validated an AG risk score based on seven modifiable lifestyle factors. Although it demonstrated moderate discriminatory power (AUC = 0.597) for a lifestyle-only screening tool, the score exhibited good calibration, clinically meaningful utility (providing net benefit within a threshold probability range of 10–60%), and exceptional operational simplicity. It can serve as a practical primary screening tool in primary care settings and population-based screening to rapidly identify individuals at high risk for AG, offering a simple and feasible risk assessment method for the primary prevention of gastric cancer.

## Data Availability

The raw data supporting the conclusions of this article will be made available by the authors, without undue reservation.
