# Supplementary material for: A lifestyle-derived risk score for chronic atrophic gastritis: development and validation in 21,008 Chinese adults
Source: Front Public Health. 2026 Apr 21;14:1784898. doi: 10.3389/fpubh.2026.1784898 (PMC13139164; doi:10.3389/fpubh.2026.1784898)
Supplement: Supplementary file 1 [file Table_1.docx]

**Supplementary Table 1.Sensitivity Analysis Using Different Cut-off Values to Define the High-Risk Group**

| **Cut-off Definition** | **OR (95% CI)** | **P-value** |
| --- | --- | --- |
| Definition of High-Risk Group |  |  |
| ≥1vs.<1 | 1.396(1.310-1.488) | <0.001 |
| ≥2vs.<2 | 2.144(2.021-2.274) | <0.001 |
| ≥3vs.<3 | 2.728(2.527-2.944) | <0.001 |

**Note:OR=odds ratio;CI=confidence interval.All models were adjusted for age and sex.The reference group in each comparison was the low-risk group defined by the corresponding cutoff point.**

**Supplementary Table 2.Sensitivity Analysis: Comparison of Different Scoring Methods**

| **Scoring Method** | **Number of Items** | **Description** | **OR** | **95%CI** | **AUC** |
| --- | --- | --- | --- | --- | --- |
| Original 7-Item Score | 7 | Included all theoretical items. | 1.487 | 1.440-1.535 | 0.583 |
| Simplified 5-Item Score | 5 | Excluded fried foods and low-frequency garlic. | 1.968 | 1.889-2.051 | 0.622 |
| Core 3-Item Score | 3 | Included smoking, alcohol consumption, and high-frequency pickled food consumption. | 2.297 | 2.189-2.412 | 0.633 |

Note: AUC values for the original 7-item score vary slightly depending on scoring method (binary vs. continuous); the value shown here (0.583) corresponds to the binary scoring approach for consistency with simplified versions.

**Supplementary Table 3.E-value Analysis Results**

| **Metric** | **Value** | **Interpretation** |
| --- | --- | --- |
| E-value for point estimate | 2.38 | The minimum strength of association required for an unmeasured confounder to explain the main result (OR=1.506). |
| E-value for CI lower bound | 2.28 | The minimum strength of association required to explain the most conservative result (OR=1.460). |
| E-value to nullify | 1.23 | The minimum strength of association required to reduce the OR to 1.0 (null effect). |
